# Semantic control deficits impair understanding of thematic relationships more than object identity

**DOI:** 10.1016/j.neuropsychologia.2017.08.013

**Published:** 2017-09

**Authors:** Hannah Thompson, James Davey, Paul Hoffman, Glyn Hallam, Rebecca Kosinski, Sarah Howkins, Emma Wooffindin, Rebecca Gabbitas, Elizabeth Jefferies

**Affiliations:** a School of Psychology, University of Surrey, UK; b Department of Psychology, University of York, UK; c Centre for Cognitive Ageing and Cognitive Epidemiology, Department of Psychology, University of Edinburgh, UK

**Keywords:** Semantic, Control, Aphasia, Stroke, Thematic

## Abstract

Recent work has suggested a potential link between the neurocognitive mechanisms supporting the retrieval of events and thematic associations (i.e., knowledge about how concepts relate in a meaningful context) and semantic control processes that support the capacity to shape retrieval to suit the circumstances. Thematic associations and events are inherently flexible: the meaning of an item changes depending on the context (for example, lamp goes with reading, bicycle and police). Control processes might stabilise weak yet currently-relevant interpretations during event understanding. In contrast, semantic retrieval for objects (to understand what items are, and the categories they belong to) is potentially constrained by sensory-motor features (e.g., bright light) that change less across contexts. Semantic control and event understanding produce overlapping patterns of activation in healthy participants in left prefrontal and temporoparietal regions, but the potential causal link between these aspects of semantic cognition has not been examined. We predict that event understanding relies on semantic control, due to associations being necessarily context-dependent and variable. We tested this hypothesis in two ways: (i) by examining thematic associations and object identity in patients with semantic aphasia, who have well-documented deficits of semantic control following left frontoparietal stroke and (ii) using the same tasks in healthy controls under dual-task conditions that depleted the capacity for cognitive control. The patients were impaired on both identity and thematic matching tasks, and they showed particular difficulty on non-dominant thematic associations which required greater control over semantic retrieval. Healthy participants showed the same pattern under conditions of divided attention. These findings support the view that semantic control is necessary for organising and constraining the retrieval of thematic associations.

## Highlights

• This manuscript compares performance on thematic and identity tasks.
• We predict that thematic tasks will require additional semantic-executive mechanisms.
• Semantic Aphasia patients show particular deficits in the thematic task in relation to the identity task, particularly weak associations.
• Healthy subjects, in an executively demanding dual-task, showed a similar pattern to the patients.
• These results support the Controlled Semantic Cognition framework, and provide evidence against the dual hub account.

## 1 . Introduction

Across our lifetime we acquire rich and varied conceptual knowledge, making it necessary to constrain retrieval so that it is focussed on only the information that is relevant for the current task or context (Badre et al., 2005, Jefferies, 2013, Noonan et al., 2010). We have knowledge about what *objects* are and the categories they belong to (e.g., *taxonomic knowledge* – identifying that an animal that barks, has a wet nose and has spots is a Dalmatian), as well as *thematic knowledge* of how objects are used and how they relate to other objects in the context of *events* (e.g., associating spoon with sugar in the context of drinking tea, even though these objects do not share physical features). This knowledge needs to be stored and accessed in a context-flexible manner. The neural organisation of these different facets of semantic cognition – both the distinction between object and event knowledge, and between conceptual representations and control processes – remains highly controversial. These are set out in two parallel lines of literature, proposing (1) two distinct storage hubs for object and event knowledge (Schwartz et al., 2011) and (2) a heteromodal conceptual hub integrating information within modality-specific spokes, plus executive-access mechanisms that shape the information that is retrieved so that it is relevant to the current task or context (Lambon Ralph et al., 2017). These accounts propose alternative roles for similar brain areas, yet there have been few attempts to directly compare them. Here we investigate whether differences between object and thematic knowledge might be partially explained in terms of their reliance on semantic control processes.

Some researchers have proposed *two separate hubs* for taxonomic and thematic knowledge, in the anterior temporal lobe (ATL) and temporoparietal cortex respectively (Binder and Desai, 2011, Kalénine et al., 2012, Kalénine et al., 2013, Schwartz et al., 2011, Solène and Buxbaum, 2016). Neuropsychological evidence for this view is provided by picture naming errors, with damage to the ATL associated with category co-ordinate or superordinate errors (e.g., apple – “fruit”) and temporoparietal damage associated with thematic errors, such as responding “nuts” to a picture of squirrel (Jefferies et al., 2008, Schwartz et al., 2011). This pattern has been argued to reflect perturbation of distinct types of knowledge following damage to dissociable areas of cortex. The two hub account has also received support from neuroimaging studies showing greater activity in posterior temporal and/or parietal regions in response to thematic judgments (de Zubicaray et al., 2013, Kalénine et al., 2009). Temporoparietal regions are consistently responsive to praxis, visual motion, actions and motor planning (Martin, 2007, Noppeney, 2008). These regions might therefore be well-placed to support the comprehension of events and thematic associations. Equally, the ATL is at the end of the ventral visual stream, and it has been previously associated with the integration of featural knowledge (Bemis and Pylkkanen, 2011, Moss et al., 2005).

However, a number of fMRI investigations have failed to find a distinction between thematic and categorical knowledge in these proposed hub regions (Jackson et al., 2015, Kotz, 2002, Sachs et al., 2008, Sass et al., 2009). Experimentally, it is difficult to entirely separate tasks on the basis of identity or thematic knowledge, since thematic tasks necessarily involve identifying what objects are, while items drawn from the same category (e.g., dog and sheep) almost always share thematic associations (Jackson et al., 2015). Moreover, an alternative hypothesis, the Controlled Semantic Cognition framework (Lambon Ralph et al., 2017), does not distinguish knowledge by its nature (thematic or taxonomic), but by its *accessibility*. By this view, the ATL is thought to form a central semantic hub encompassing multiple aspects of knowledge (Lambon Ralph et al., 2017, Patterson et al., 2007). Patients with semantic dementia (SD), who have relatively focal degeneration of the ATL bilaterally (Mummery et al., 2000), show highly consistent errors for the same concepts across different tasks – including across object matching and thematic matching paradigms (Bozeat et al., 2000, Hoffman et al., 2013, Jefferies and Lambon Ralph, 2006), consistent with degradation of core conceptual knowledge that encompasses both the physical and associative features of items. The semantic deficit in SD erodes the distinction between specific concepts first, such that patients can no longer distinguish a Dalmatian from other breeds of dog; but can identify that this item is an animal (Hodges and Patterson, 2007, Mummery et al., 2000, li et al., 2006). ATL is thought to integrate modality-specific features, allowing deep conceptual similarities and distinctions to be extracted (the "hub and spoke model"; Patterson et al., 2007; Rogers et al., 2006; Rogers et al., 2004). There is also growing evidence from distortion-corrected and distortion-limiting fMRI methods and transcranial magnetic stimulation studies that the ventral ATL is involved in multimodal semantic processing in healthy participants, in line with this view (Binney et al., 2010, Pobric et al., 2010, Visser et al., 2012).

**Fig. 1:**
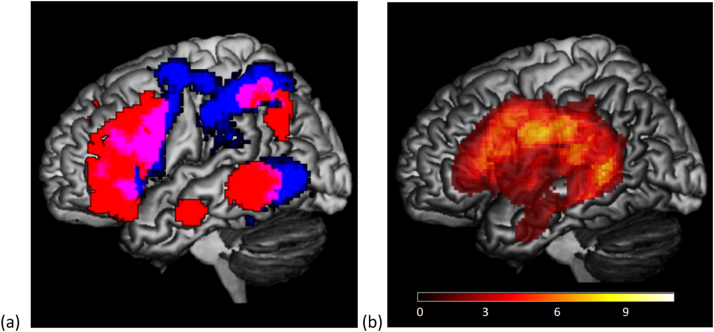
(a) overlap of semantic control regions (red), taken from Noonan et al. (2013); and ‘action’ regions (blue), taken from an automated meta-analysis of 708 studies using Neurosynth (http://neurosynth.org/). The overlap of the control and action regions is in pink (in pMTG, anterior IPL, premotor cortex and posterior IFG). (b) SA lesion overlay map showing areas of maximum overlap (11 patients in total). (For interpretation of the references to color in this figure legend, the reader is referred to the web version of this article.)

The Controlled Semantic Cognition account proposes that semantic knowledge interacts with control processes to allow appropriate semantically-driven thoughts and behaviour (Lambon Ralph et al., 2017, Jefferies, 2013). Consequently, when distinctions between types of knowledge (thematic or taxonomic) occur, these may stem from differences in accessibility or control requirements. Thematic judgments are contextually-guided: there are diverse associations to any given concept and thus it is necessary to shape retrieval to focus on the specific links that are relevant to a particular situation: for example, the word lamp may be associated with bicycle but also with reading, depending on the circumstances. A common network of brain regions including left inferior frontal gyrus (IFG) and posterior middle temporal gyrus (pMTG) is implicated in situations in which semantic cognition is relatively controlled, i.e., during the retrieval of ambiguous word meanings and weak semantic relationships (Davey et al., 2016, Noonan et al., 2013, Whitney et al., 2011), and also in understanding events, actions and thematic associations (de Zubicaray et al., 2013, Kalénine et al., 2013, Mirman and Graziano, 2012). For example, a recent study found overlapping voxels within both left IFG and pMTG for contrasts examining action understanding and semantic control when these were manipulated within a single study in the same participants (Davey et al., 2015b; see also Fig. 1 below). Left IFG and pMTG are key components of a large-scale network activated by diverse manipulations of semantic control demands in an activation-likelihood meta-analysis (Noonan et al., 2013) and the co-activation of these regions has also been observed in individual studies (Davey et al., 2016). Moreover, TMS to both regions produces an equivalent disruption of tasks requiring semantic control but not more automatic semantic association judgements (Davey et al., 2015a, Whitney et al., 2011). Together, these findings suggest that related and overlapping brain networks might support both semantic control and the retrieval of thematic or event knowledge. This overlap might reflect the inherent flexibility of thematic associations, actions and events: when making judgements to these kinds of stimuli, there is a need to flexibly prioritise different features within the long-term semantic store depending on the context in which concepts occur. Control processes might help to stabilise weak yet currently-relevant interpretations in these kinds of tasks: for example, they may promote particular associations that are relevant to the link with a target word, or potential action features of objects that allow an item to be used in a particular way, which is suited to the context. In contrast, semantic tasks focussed on object identity and categorical distinctions may be more constrained by sensory-motor features (e.g., lamps have a bright light and they can be categorised with other objects with this property, such as torch): these core features arguably change less across contexts (although such features are not always present, and physical features such as size and colour can also be variable across exemplars).

Patients with Semantic Aphasia (SA), show multimodal semantic control deficits that appear to reflect difficulty using knowledge in a task- and context-appropriate way (Jefferies and Lambon Ralph, 2006, Rogers et al., 2015). This contrasts with Semantic Dementia patients (SD), who show evidence of disintegrating stored knowledge (Jefferies and Lambon Ralph, 2006). The ventral ATL multimodal ‘hub’ region which is the focus of atrophy in SD is rarely affected in SA patients, as it is anatomically protected from stroke – receiving a dual blood supply (Phan et al., 2005, Phan et al., 2007). Typically, SA patients have damage to LIFG and/or posterior temporoparietal cortex (Noonan et al., 2010, Thompson et al., 2015). They show pronounced deficits on tests of object use, event understanding and knowledge of thematic relations (Corbett et al., 2009a, Corbett et al., 2009b, Corbett et al., 2009a, Corbett et al., 2009b, Corbett et al., 2011, Jefferies and Lambon Ralph, 2006). However, they are also sensitive to the control demands of semantic tasks beyond thematic judgements: for example, they select highly-associated distracters in synonym judgement tasks (e.g., they choose cake instead of matching piece with slice) and they show impaired performance on identity matching tasks when these tasks generate strong competition through the repeated presentation of the same items as targets and distracters on different trials (Gardner et al., 2012, Jefferies et al., 2007, Thompson et al., 2015). In line with the executive nature of their deficit, they are sensitive to cues that reduce the requirement for internally-generated constraint on semantic retrieval and to miscues that direct attention inappropriately towards irrelevant aspects of knowledge, and they struggle to identify semantic relationships where the relationship is distant or in the absence of strongly overlapping features (e.g., Noonan et al., 2010; Corbett et al., 2011). They additionally show deficits in domain-general executive control parallel to their semantic impairment (Jefferies and Lambon Ralph, 2006).

In summary, studies have provided some evidence that the neurocognitive components that support understanding of events and thematic associations are partially overlapping with control processes within semantic cognition, which tailor retrieval to suit the circumstances. In this study, we tested the hypothesis that semantic control and thematic knowledge are directly related, by assessing whether reduced semantic control causes greater impairments for tasks tapping thematic knowledge than object identity knowledge, both in patients with SA who have well-characterised deficits of semantic control and in healthy participants under conditions of divided attention. We employed similar picture-word matching tasks across thematic and identity tasks – requiring retrieval of thematic relationships based on the interaction of objects in an event context (e.g., dog and beach; champagne and racing car) or the identification of these objects (e.g., picture of dog with its name Dalmatian). We compared weak and strong thematic relationships, and identity matching at different levels of specificity. Weak associations (e.g., dog with beach) are harder to identify than strong associations, such as dog-bone and beach-sand, since additional control over semantic retrieval is needed to focus on relevant conceptual information and to ignore strong but irrelevant associations (Badre et al., 2005). We additionally had two identity tasks: specific-level matching (e.g., picture of dog with word dalmatian) and superordinate identification (dog with word animal). The direction of difficulty is less clear-cut in this task: superordinate labels are more frequent, since they describe a broader range of concepts. However, although specific level matching requires knowledge of lower frequency words and finer-grained conceptual differentiation (and thus might be more difficult for participants without a control deficit), superordinate labels encompass diverse objects, which are likely to include features not shared with the probe on a given trial. In this way, superordinate terms have greater *contextual diversity*; they are used in a greater range of contexts, to mean slightly different things (Hoffman et al., 2011). The word animal may refer to pets, or badly-behaving humans, for example, while dalmatian always refers to a specific type of dog. For this reason, superordinate matching may require more control than specific matching, in line with reports of reverse specificity effects in dysexecutive patients (Humphreys and Forde, 2005).

If SA patients have disruption to a store of thematic knowledge (Schwartz et al., 2011), we would predict deficits in the thematic task regardless of associative strength. In contrast, if their difficulties on thematic matching tasks are largely related to the increased control demands of these decisions, we would expect greater impairment of thematic than object matching tasks in general, but particularly deficits on thematic trials probing weak associations when there is a greater requirement to bias retrieval away from knowledge dominant in the long-term conceptual store towards weaker conceptual patterns. We would also anticipate some degree of deficit on identity matching trials, given these judgements require control over semantic retrieval to some extent. To further test these hypotheses, we considered whether SA-like deficits could be induced through the use of a secondary task in healthy volunteers. This method has previously been used to disrupt comprehension of ambiguous words, mimicking the pattern in SA (Almaghyuli et al., 2012). Participants therefore performed the semantic tasks concurrently with a secondary task that either required a high degree of executive control over the retrieval of memory representations (1-back) or little executive control (counting numbers sequentially). This approach could provide further support the Controlled Semantic Cognition framework, as healthy controls do not have a deficit of thematic knowledge per se and would have no reason to find thematic tasks problematic under dual-task conditions except if these judgements load executive mechanisms.

## 2 Method

### 2.1 Participants

11 stroke aphasia patients (mean age = 66 years, s.d. = 7.8; mean age of leaving education = 16.3 years, s.d. = 1.3) were recruited through stroke and aphasia groups across Yorkshire and Manchester, UK. Patients were native English speakers and had chronic aphasia from a left hemisphere cardiovascular accident (CVA) at least 1 year previously. They were not selected to have either semantic control deficits or thematic deficits, but instead to show multimodal impairment on a range of standard semantic assessments.

As a group, the patients were somewhat milder than those previously described (Jefferies and Lambon Ralph, 2006, Thompson et al., 2015), although all showed deficits in at least one of four tasks from the Cambridge Semantic Battery (CCTp, CCTw, word-picture matching or naming). 4/11 showed impairment on both the picture and word version of a semantic association task (the Camel and Cactus task, CCT, Bozeat et al., 2000). The remaining 7 cases showed deficits on more demanding tasks: namely impairment on verbal tasks such as synonym judgement and/or semantic matching for ambiguous words, plus deficits on a non-verbal picture-matching task probing object use (Corbett et al., 2011, Noonan et al., 2010) (see Table 2, Table 3). In this way, they met the criteria for semantic aphasia used by Jefferies and Lambon Ralph (2006), since every case showed evidence of a multimodal semantic deficit.

10 healthy age-matched controls (mean age = 71.7 years, s.d. = 7.7, mean age of leaving education = 18.2 years, s.d. = 2.7), who had no neurological impairment, were compared with the patients. There were no significant differences between these groups in age or education (t ≤ 1.898, p ≥ .076).

### 2.2 Lesions

Structural MRI scans were obtained for all cases. An overlay of lesion maps of all patients was created from automated lesion identification (Seghier et al., 2008) and is displayed in Fig. 1b. The highest lesion overlap in the SA group was seen in posterior frontal, inferior parietal and posterior temporal cortex, and thus these patients had damage to brain regions typically implicated in action/event understanding and semantic control shown in Fig. 1a. 3/11 patients had damage restricted to temporo-parietal regions (TP-only). The other 8/11 patients had damage extending to left inferior frontal regions (PF+). Lesions were characterised by manual lesion tracing using Damasio templates (Damasio and Damasio, 1989). Table 1 displays details of the patients’ lesions, focusing on regions of interest in temporal, parietal and frontal cortex. The inferior anterior temporal lobe, implicated in amodal semantic representation and atrophied in semantic dementia, was spared in all cases (Binney et al., 2010).

**Table 1:** Patient lesions.

Patient	Lesion subgroup	Lesion size^a^ (% of template damaged	Years since CVA	Upper limb Hemiplegia?	DLPFC		orbIFC	trIFC	opIFG	STG	MTG	ITG	FG	POT	AG	SMG	TP
					BA9	BA 46	BA 47	BA 45	BA 44	BA 22	BA 21	BA 20	BA 36	BA 37	BA 39	BA 40	BA 38
TK	TP-only	4.8	?	?						1					1	2	
KS	TP-only	2.4	5.5	✗						1	2			2			
RDE	TP-only	.6	3	✗										1	1		
NNF	PF+	12.4	8.5	✓			2	1	1	2	1			2	1	2	
NGW	PF+	7.8	23	✓					1	2				2		1	
SSR	PF+	14.4	6.5	✓			2	1	2	2				1		1	
NNZ	PF+	4.4	4.5	✓					1	1	1			1			
NTG	PF+	12.4	8	✓			1			2				2		1	
NHY	PF+	6.5	13	✓				1	2					1	1	1	
LHN	PF+	14.8	7	✓	2		2	2	2	2						1	
HNA	PF+	12	9	✓	1	1		1	1	2				1	1	2	

Quantification of lesion: 2 = complete destruction/serious damage to cortical grey matter; 1 = partial destruction/mild damage to cortical grey matter. PF+ = lesions extending to prefrontal cortex. TP-only = lesions restricted to temporoparietal cortex. Anatomical abbreviations: DLPFC = dorsolateral prefrontal cortex; orbIFG = pars orbitalis in inferior frontal gyrus; trIFG,= pars triangularis in inferior frontal gyrus; opIFG = pars opercularis in inferior frontal gyrus; TP = temporal pole; STG = superior temporal gyrus; MTG = middle temporal gyrus; ITG = inferior temporal gyrus; FG = fusiform gyrus; POT = posterior occipitotemporal area; SMG = supramarginal gyrus; AG = angular gyrus. ^a^Lesion size was estimated by overlaying a standardised grid of squares onto each patient's template and working out the percentage of squares damaged relative to the complete undamaged template.

### 2.3 Neuropsychological assessment

A battery of tasks was used to examine executive function and semantic performance.

Non-semantic tasks included the following: (1) *Forward and backward digit span* (Wechsler, 1987), to assess auditory working memory; (2) *Ravens Coloured Progressive Matrices test* (RCPM: Raven, 1962), to assess non-verbal reasoning; (3) *The Visual Object and Space Processing battery, VOSP* (Warrington and James, 1991): Dot Counting, Position Discrimination, Number Location, and Cube Analysis subtests; (4) *Elevator Counting*, from the Test of Everyday Attention (TEA; Robertson et al., 1994), required auditory tones to be counted with and without distraction; (5) *The Brixton Spatial Rule Attainment task* (BSRA: Burgess and Shallice, 1997), involved making predictions about the movement of a dot, based on patterns that it showed across trials, and then adapting these predictions when the pattern changed; (6) *Trail making* required participants to draw a line between letters and numbers in order, in an easy condition (e.g., 1–2–3…) and difficult condition (e.g., 1-A-2-B-3-C…, Reitan, 1958).

Semantic tasks included the following: (1) *64-item Cambridge semantic battery* (Bozeat et al., 2000), which presented the same items in multiple tasks: (i) *spoken word-picture matching* (WPM), (ii) *picture naming*, (iii) *picture Camel and Cactus Test*, (CCT); (iv) *word CCT*. The CCT involved identifying which of four pictures/words was most associated with a probe picture/word (e.g., camel with cactus, rose, tree or sunflower?). (2) *96-item synonym judgement task* (Jefferies et al., 2009). A probe word was matched to a synonym target presented with two unrelated distractors. This had 96 items in two frequency bands (high and low) and three imageability bands (high, medium and low), producing sixteen trials in each of the six frequency-by-imageability conditions (see Jefferies et al. (2009)). The words were printed and also read aloud to the participants. Responses were untimed. (3) *Category fluency*, requiring participants to think of as many items from a particular category as they could (categories were: animals, fruit, birds, breeds of dog, household objects, tools, vehicles and types of boat) and (4) *Letter fluency*, which required participants to produce as many words as possible within one minute which begin with a certain letter (F, A, F, A, S).

Semantic control tasks included the following: (1) Ambiguous words (Noonan et al., 2010) using polysemous words to test comprehension of dominant (e.g., pen-pencil) and subordinate (e.g., pen-pig) meanings of words. There were 30 words, presented in both dominant and subordinate conditions, and the target was presented amongst 3 unrelated distractors which were the same across conditions. (2) 37-item object use task, assessing canonical and non-canonical uses of everyday objects (Corbett et al., 2011), such as swatting a fly with a fly swat (canonical) or rolled up newspaper (non-canonical). The target was presented with 3 related and 2 unrelated foils, and these foils were identical across the canonical/non-canonical conditions. (3) Synonym task manipulating distractor strength (Noonan et al., 2010, Samson et al., 2007), which required patients to match words according to their meaning (e.g., piece and slice) while ignoring a thematic distractor (cake). The thematic distractor was either strongly or weakly related to the target; e.g., ‘cake’ (strong distractor for piece) and ‘letter’ (a weak distractor for ‘reply’ with ‘answer’). There was also an unrelated distractor and therefore three response options. This test consisted of 84 items, 42 weak and 42 strong distractors.

## 3 Results

Patients showed abnormal performance on a range of semantic and non-semantic executive tasks (see Table 2). They were impaired at basic semantic tasks across modalities – some of which tapped knowledge of object identity (e.g., picture naming and word-picture matching) and some of which were about events and relationships (e.g., Camel and Cactus Tasks in picture and word versions). They were also impaired on previously published tasks manipulating semantic control, displayed in Table 3. They showed greater deficits in retrieving non-canonical relative to canonical uses of objects. Similarly, the majority of patients showed substantial differences between the comprehension of subordinate vs. dominant meanings of ambiguous words: in both of these tasks, the SA patients had deficits in flexible semantic retrieval – i.e., they found it hard to focus on non-dominant yet currently-relevant aspects of meaning when trying to retrieve associations. They were also influenced by distractor strength in synonym judgement (i.e., matching words with similar meanings, as opposed to words linked by a thematic association). This demonstrates that they found it hard to ignore currently-irrelevant yet strong associations even when the task did not require access to thematic knowledge.

**Table 2:** Background neuropsychological, semantic and executive test performance for the patient sample.

**Patient**	**Digit span**	**Backward digit span**	**RCPM**	**VOSP**	**TEA – no distraction**	**TEA – distraction**	**Brixton**	**Trails A**	**Trails B**	**Spoken WPM**	**Naming**	**CCTp**	**CCTw**	**Synonyms**	**Category fluency**	**Letter fluency**
**Max**	7	7	36	50	7	10	54	24	23	64	64	64	64	96	–	–
**Control mean SD**	6.8 (.6)^c^	5.6 (1.0)^d^	32.9 (2.4)^a^	48.1 (3.5)	6.6 (1.2)	8.2 (2.8)	32.8 (8.6)	24 (0)^b^	21.6 (2.1)^b^	63.7 (.5)	62.3 (1.6)	58.9 (3.1)	60.7 (2.06)	94.4 (1.2)	95.7 (16.5)	44.2 (11.2)
**Normal cut-off**	5.6	3.7	28.2	41.3	4.2	2.6	28		17.4	63	59	52.7	56.6	92	62	21
**NNF**	2*	0*	31	34*	6	1*	18*	23	16*	60*	19*	45*	29*	71*	15*	2*
**NGW**	5*	2*	24*	42	5	1*	26*	24	12*	64	50*	56	53*	74*	26*	2*
**SSR**	NT	NT	34	47	7	7	31	24	23	52*	3*	54	57	87*	NT	NT
**NNZ**	5*	3*	21*	45	5	2*	31	24	19	64	56*	53	61	78*	80	16*
**NTG**	3*	NT	22*	NT	NT	NT	36	NT	NT	16/16 ^f^	10/16 ^f^	57	56*	NT	NT	NT
**NHY**	4*	0*	30	49	NT	NT	23*	23	5*	62*	50*	57	52*	76*	26*	6*
**LHN**	4*	2*	29	49	5	3	7*	22	23	62*	61	44*	43*	59*	NT	NT
**HNA**	NT	NT	31	42	2*	1*	21*	19	2*	63	1*	31*	39*	57*	NT	NT
**TK**	3*	3*	25*	44	6	2*	30	24	0*	64	12*	54	48*	69*	19*	5*
**KS**	8	4	31	42	9	5	29	23	7*	46*	21*	44*	42*	81*	24*	19
**RDE**	5*	NT	21*	NT	NT	NT	5*	24	3*	15/16 ^f^	13/16 ^f^	9/25 ^f^	49*	90*	NT	NT

RCPM = Raven's Coloured Progressive Matrices (Raven, 1962); VOSP = Visual Object and Space Perception battery (Warrington and James, 1991) 6, 7, 8, 9; TEA = Test of Everyday Attention (Robertson et al., 1994); Trails = Trail Making Test (Reitan, 1958); Brixton = Brixton Spatial Rule Attainment Task (Burgess and Shallice, 1997); Spoken Word-Picture Matching (WPM) from the Cambridge Semantic Battery (Bozeat et al., 2000); Synonym judgment (Jefferies et al., 2009); Environmental Sounds Test (Bozeat et al., 2000); CCT = Camel and Cactus task in picture and written word forms (Bozeat et al., 2000); PPT = Pyramids and Palm Trees task in picture and written word forms (Howard and Patterson, 1992); category fluency = 8 categories; letter fluency = ‘F′, ‘A′, ‘S′; NT = not tested; norms taken from published data sets unless otherwise stated. Healthy aged-matched controls were tested at York, cut offs were 2 SD below the mean. Slightly different numbers of healthy subjects took part in each task, as follows: ^a^ = 21; ^b^ = 14; ^c^ = 17; ^d^ = 10; ^e^ = 16. ^f^ - RDE and NTG only completed a subset of items from the Cambridge Semantic battery before withdrawing from the study for health reasons.

**Table 3:** Performance on semantic control tasks.

	**Object use task**			**Ambiguity task**			**Synonym task**		
**Patient**	**Canonical**	**Non-canonical**	**Overall score**	**Dominant**	**Non-dominant**	**Overall score**	**Weak distractor**	**Strong distractor**	**Overall score**
**Max**	37	37	74	30	30	60	*42*	*42*	*84*
**Control mean, SD**	NT	33.6 (2.2)		29.5 (.5)	28.9 (.6)	58.4 (.7)	41.5 (.5)	39.9 (2.2)	81.4 (2.6)
**Normal cut-off**		29.2		*28.4*	*27.6*	*56.2*	40.4	35.4	76.1
**NNF**	29	14***	43	24***	14***	38***	29***	13***	42***
**NGW**	35	21***	56	22***	14***	36***	24***	20***	44***
**SSR**	33	22**	55	27**	19***	46***	31***	30**	61***
**NNZ**	37	26*	63	28*	21***	49***	28***	22***	50***
**NTG**	NT	NT	NT	NT	NT	NT	NT	NT	NT
**NHY**	35	22**	57	26***	17***	43***	30***	23***	53***
**LHN**	31	13***	44	18***	9***	27***	23***	12***	35***
**HNA**	32	14***	46	22***	11***	33***	25***	15***	40***
**TK**	37	27*	64	22***	21***	43***	NT	NT	NT
**KS**	30	12***	42	21***	13***	34***	34***	21***	55***
**RDE**	NT	NT	NT	26***	21***	47***	NT	NT	NT

* ≤ .05, ** ≤ .01, *** ≤ .001 two-tailed ‘Singlims’ (Crawford and Garthwaite, 2002), which uses a modified t-statistic to examine whether an individual is significant below a control group, taking into account group size and standard deviation. Object use task with canonical and non-canonical subsections (Corbett et al., 2011). Ambiguity with dominant and non-dominant subsections (Noonan et al., 2010), and synonym task with strong and weak distractors (Noonan et al., 2010). Control means reported are previously published.

### 3.1 Identity and thematic matching in SA

**Fig. 2:**
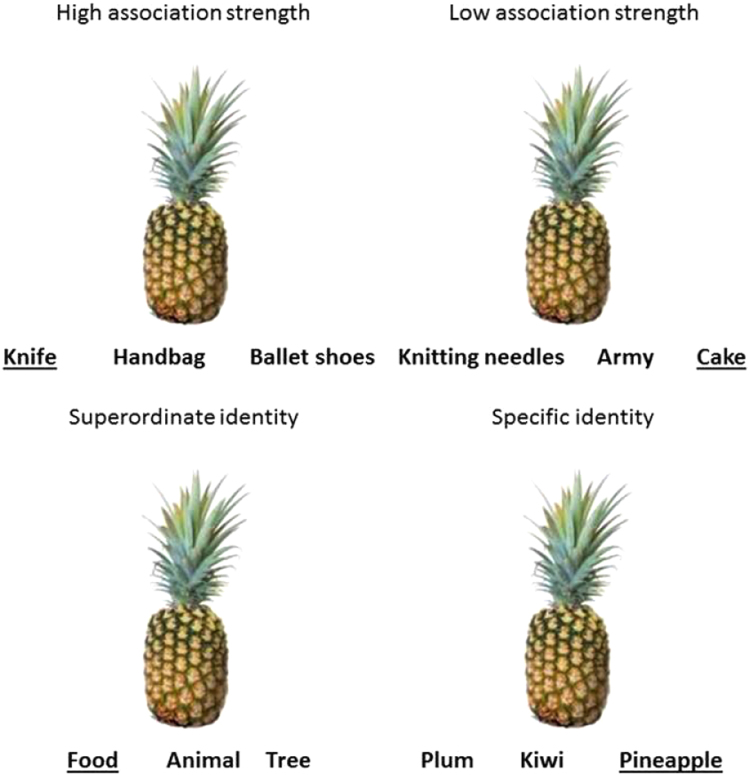
Examples of thematic and identity matching trials for both versions. The target word is underlined in each case.

There were two semantic tasks (identity and thematic matching), each with two versions, shown in Fig. 2. For identity matching, participants matched a photograph of a probe object with a more general, superordinate category label or with a more specific name, requiring finer-grained semantic analysis. For thematic matching, participants were instructed to select the word which had the strongest link to the picture: easier decisions were based on strong thematic associations; while harder decisions were based on weak thematic associations.

## 4 Method

All of the judgements involved picture-word matching with three written options (3AFC), with stimuli listed in supplementary materials. The final set of matched words was identified using the program Match, detailed below (Van Casteren and Davis, 2007). Trials that did not permit a match were removed prior to analysis, leaving 58 trials per condition, and a total of 232 items, with an extra 12 items in a practice block. The match process meant not all probes were the same across conditions. Picture probes were colour photographs. All were resized to 200 × 200 pixels whilst maintaining the aspect ratio. Participants then decided which of three words went with the picture. In the superordinate identity matching trials, target words corresponded to the general category label (e.g., food, clothes, vehicle, repeated across trials) and distracters were other superordinate labels. In the specific matching trials, target words were the most specific label commonly known by participants, and distracter words were specific level names from the same basic category, e.g., bulldog, terrier, dalmatian. For thematic matching trials, distracter items were unrelated words (Davey et al., 2015a). All words were nouns.

Psycholinguistic matching was achieved using familarity and imagablity statistics taken from the MRC Psycholingustic Database (Coltheart, 1981; extracted using the program N-Watch; Davis, 2005), supplemented by additional ratings from 22 participants not included in the experiment. Statistics for target words are shown in Table 4 (although the superordinate condition is omitted since these labels were repeated across multiple trials). Trials were matched across conditions, with independent samples *t*-tests reported in Table 4. Familiarity, word length and imageability were matched across specific identity matching and thematic trials, and between high and low-strength thematic trials. Association strength (estimated using pairwise comparison in the latent semantic analysis database (Dumais, 2005, Landauer et al., 1998), on the basis of the co-occurrence of the words in text) was significantly higher for strong than weak associations.

**Table 4:** Word statistics for the trials used in both experiments.

Condition	Word length		t-statistic	Imageability		t-statistic	Familiarity		t-statistic	Association strength		t-statistic
	Mean	S.D.		Mean	S.D.		Mean	S.D.		Mean	S.D.	
Identity – specific	7.07	2.22	t < 1^b^	4.71	.88	t < 1^b^	6.00	.85	t < 1^b^			–
Thematic – high strength	6.97	2.11	t < 1^a^	4.78	1.03	t < 1^a^	6.00	.78	t(144) = 1.685, p = .095^a^	.25	.19	t(98) = 2.588, p = .011^a^
Thematic – low strength	6.98	2.22		4.73	.92		6.22	.58		.17	.15	

Numbers reflect statistics for target words. ^a^ = *t*-test comparing high and low thematic strength. ^b^ = *t*-test comparing specific-identity trials with both thematic tasks combined. Word length = number of letters. Imageability = on a 7-point scale, where 7 indicates highly imageable. Familiarity = on a 7-point scale, where 7 indicates highly familiar. Association strength from Latent Semantic Analysis (Landauer et al., 1998), which extracts similarities of words (in this case, probe and target) based on the statistical likelihood of co-occurring in text (max = 1). Identity – superordinate trials are not included since we opted to test the same items at a specific and superordinate level, and thus we repeated 10 category labels (food, sports equipment, animal, tree, footwear, clothes, vehicle, instrument, weapon and household item) across these superordinate trials.

Trials were presented in mini-blocks, each containing 10 trials from one of the conditions. If required, the experimenter read out the words to the patient. The order of trials within a block was randomized across subjects. Patients did the experiment in two parts, completing other neuropsychological tests in between. Participants made their responses by pressing 1, 2, or 3 on the keyboard to indicate which of three response options matched the picture. The researcher pressed these buttons for two of the more impaired patients (NNF and HNA), who responded by pointing.

## 5 Results

**Fig. 3:**
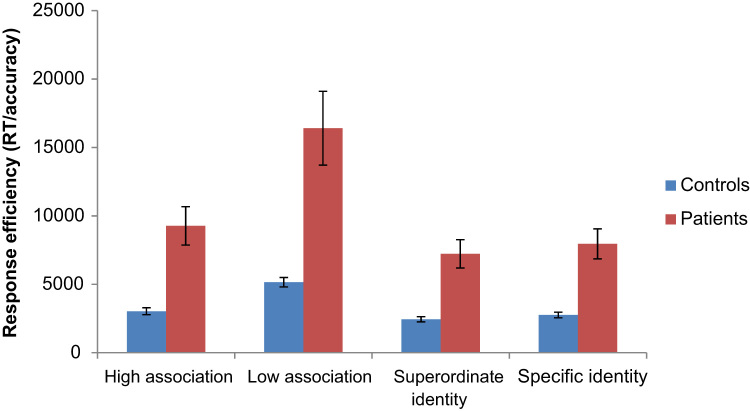
Response efficiency for both healthy controls and patients. Error bars represent the standard error.

For both patients and controls, incorrect trials and outlying responses more than two standard deviations away from an individual's mean for that condition were removed prior to RT analysis. Behavioural results are shown in Table 5. We also examined response efficiency (reaction time divided by accuracy), which is displayed in Fig. 3.

**Table 5:** Behavioural results for the healthy controls and patients.

	Reaction time		Accuracy	
Condition	Mean (milliseconds)	S.D.	Mean (proportion correct)	S.D.
*Healthy controls*				
High thematic strength	3002.58	845.66	1.00	.01
Low thematic strength	4956.50	1136.15	.96	.02
Superordinate identity	2354.14	657.07	.97	.03
Specific identity	2731.50	659.25	.98	.01
*Patients*				
High thematic strength	8109.32	3180.45	.90	.08
Low thematic strength	12281.23	6418.16	.76	.11
Superordinate identity	6509.20	2600.06	.92	.07
Specific identity	6830.92	2270.43	.89	.09

### 5.1 Thematic vs. identity judgements

Table 6 shows contrasts between thematic and identity matching. The thematic task was hardest overall, and patients were also more impaired on this task, leading to a significant interaction between judgement type and group in response efficiency and accuracy (and with a marginal effect for response time).

**Table 6:** ANOVAs showing difference between tasks.

	Response efficiency (RT/accuracy)		Accuracy		Reaction time	
	F	Sig.	F	Sig.	F	Sig.
Group	20.481	< .001	30.735	< .001	23.081	< .001
Judgment	23.496	< .001	10.494	.004	24.630	< .001
Group by judgement	7.301	.014	14.578	.001	4.249	.053

### 5.2 Strength of association

Table 7 shows analysis of the effect of the strength of association within the thematic task. Weak thematic associations were more difficult to retrieve, particularly for the patients, giving rise to a significant association strength by group interaction in efficiency and accuracy.

**Table 7:** ANOVAs for strength of association.

	Response efficiency (RT/accuracy)		Accuracy		Reaction time	
	F	Sig.	F	Sig.	F	Sig.
Group	16.515	.001	29.472	< .001	16.879	.001
Strength	39.039	< .001	44.034	< .001	24.526	< .001
Group by strength	11.479	.003	15.993	.001	3.312	.085

### 5.3 Specificity effects

We then compared specific and general-level matching within the identity matching task. Whilst there was no overall specificity effect, there was a group by specificity interaction for response efficiency and accuracy (Table 8). Patients showed no effect of specificity (t < 1), where controls showed the expected processing advantage for superordinate compared to specific identity matching in RT: (t(9) = 2.825, p = .020), and response efficiency (t(9) = 2.457, p = .036).

**Table 8:** ANOVAs for specificity.

	Response efficiency (RT/accuracy)		Accuracy		Reaction time	
	F	Sig.	F	Sig.	F	Sig.
Group	26.477	<; .001	19.759	<; .001	31.709	<; .001
Specificity	.780	.388	.179	.677	1.165	.294
Group by specificity	11.479	.003	15.993	.001	3.312	.085

### 5.4 Subset of items matched for difficulty

**Fig. 4:**
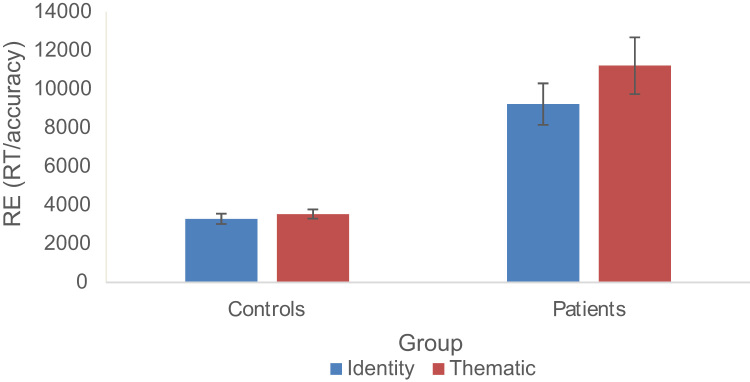
Matched-difficulty subset of items. Error bars reflect standard error of mean.

Given the overall difference in response difficulty for the identity and thematic tasks, we selected a subset of 70/116 items that were matched on response efficiency in healthy controls. This included the ‘hardest’ identity trials and ‘easiest’ thematic trials, collapsing across specificity and strength of association (41 specific-level and 29 superordinate-level identity-matching items; 54 strong associations and 16 weak associations from the thematic task). These are shown in Fig. 4.

Paired-samples *t*-tests confirmed no difference in response efficiency between thematic and identity tasks for controls: t(9) = 1.449, p = .181. For patients, however, responses were more efficient in the identity task than the thematic task (t(10) = 2.604, p = .026), consistent with the view that the patients with SA are impaired at a task in which semantic retrieval need to be flexibly tailored to suit the context.

### 5.5 Individual patient analysis

We tested whether the effects of thematic associative strength and specificity were greater in individual patients than would be expected from the distribution of test scores seen in the controls using the revised standardised difference test (RSDT; Crawford et al., 2010). This test uses a modified *t*-statistic to compare individual patients with a control group, taking into account the variability of the control data and the sample size. Results are shown in Table 9.

**Table 9:** Task manipulation effects in individual patients.

	**Reaction time (RT)**						**Accuracy**						**Response efficiency (RT/accuracy)**					
	Association strength			Identity			Association strength			Identity			Association strength			Identity		
	Strong	Weak		Super ordinate	Specific		Strong	Weak		Super ordinate	Specific		Strong	Weak		Super ordinate	Specific	
**NNF**	10636	14966		11875	9103	***	.93	.72	*	.78	.97	**	11437	20787	***	15225	9384	***
**NGW**	10327	17896	***	7464	9486	**	.88	.78	**	.97	.84	***	11735	22943	***	7695	11293	***
**SSR**	4698	7574		4297	4723		.97	.91		1	.88	***	4844	8323		4297	5367	
**NNZ**	6948	9809		4480	6211	**	1	.81	***	.98	.95		6948	12109	*	4571	6538	*
**LHN**	8253	8447	***	5911	5680		.81	.55	*	.97	.81	***	10189	15358		6094	7012	
**HNA**	7115	8580	**	7842	6376	**	.91	.64	**	.84	.95		7819	13406	*	9335	6712	***
**NTG**	4629	7423		3889	3952		.95	.83		.83	.97	*	4873	8944		4686	4074	
**NHY**	6847	15249	***	4594	6663	**	.97	.91		.97	.93	*	7058	16757	***	4736	7164	**
**RDE**	10050	11835	**	9249	7005	***	.91	.79	*	.91	.9	*	11044	14981		10164	7783	***
**TK**	4734	5577	*	3977	4652		.9	.71		.97	.91	**	5260	7855		4100	5112	
**KS**	14964	27740	***	8023	11290	***	.72	.71	***	.93	.66	***	20784	39070	***	8627	17106	***

RSDT (Crawford et al., 2010) using two-tailed probability, to see if patient performance differed significantly from the pattern of performance observed in the control group.

* <; .05.

** ≤ .01.

*** ≤ .001.

All participants were slower and less accurate on the weak compared with strong thematic association strength, and this difference was significantly larger than controls in either RT, accuracy or response efficiency in all but 2 patients (SSR and NTG). For the identity matching task, results were more mixed with many patients showing better performance for superordinate trials (the same direction as controls but with a steeper gradient; NGW, SSR, NNZ, LHN, NHY, TK, KS) and a few showing better performance for specific trials, i.e., a reverse specificity effect (NNF, HNA, NTG, RDE). It was only the most severe SA cases who showed significant reverse specificity effects, while the more mildly impaired cases (overall task accuracy > 90%) showed an exaggeration of the normal pattern.

### 5.6 Lesion location

Although many of the patients in the group had large left-hemisphere lesions, it was possible to split the group into those with and without prefrontal lesions (PF+ and TP-only patients). Logistic regression was used to examine effects of lesion location on accuracy for each task. We entered the following variables into the model: patient ID, lesion location, task, difficulty, lesion location by task, lesion location by difficulty, task by difficulty and lesion location by task by difficulty. We then ran the same regression model including each task separately (without the task variable). This is shown in Table 10.

**Table 10:** Logistic regression of lesion location on performance.

	**All data**		**Identity task**		**Thematic task**	
	Wald	p	Wald	p	Wald	p
**Patient**	73.456	<; .001	33.210	<; .001	53.594	<; .001
**Lesion**	< 1	n.s.	< 1	n.s.	< 1	n.s.
**Task**	3.743	.001				
**Difficulty**	10.200	.001	10.381	.001	6.259	.012
Difficulty * task	1.155	n.s.				
Lesion * task	3.786	.052				
Difficulty * lesion	6.598	.010	6.791	.009	4.339	.037
Difficulty * lesion * task	10.506	.001				

**Fig. 5:**
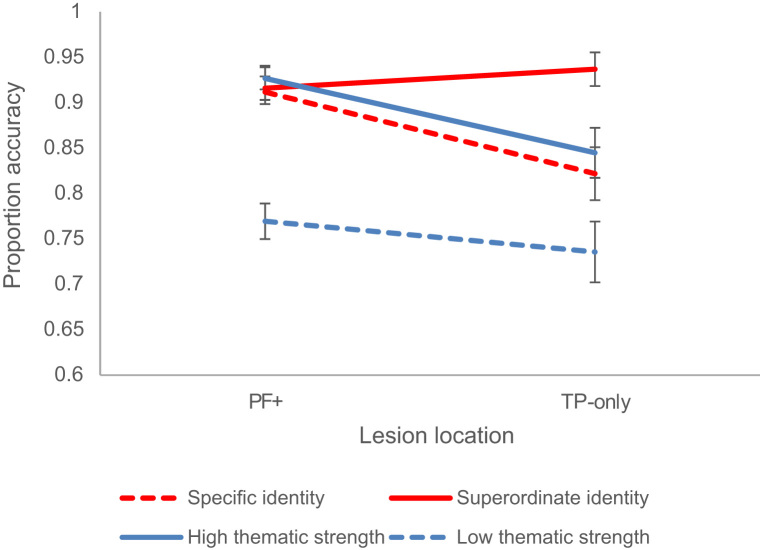
Performance according to lesion location.

We found no main effect of lesion location in any of the models. There were some important interactions with lesion location, however, displayed in Fig. 5. In the identity matching task, ‘reverse’ specificity effects (specific > superordinate) were found in some of the most impaired PF+ patients (although other PF+ patients showed the normal pattern; superordinate > specific). This meant that across all PF+ patients, there was no effect of specificity. In contrast, all the TP-only patients showed the normal superordinate > specific pattern, and consequently the standard effect of specificity was greater in the TP-only group. In the thematic task, the PF+ group showed a stronger effect of strength of association than the TP-only group, although this effect was relatively subtle.

## 6 Summary

SA cases showed a bigger effect of strength of association in the thematic task, compared to controls. They also showed greater impairment in thematic than identity matching, consistent with the hypothesis that thematic matching requires more semantic control than identity-matching, especially when the relevant associations are not dominant for particular objects. On a subset of trials in which difficulty was matched across the two tasks in the control group, the patients continued to show more impairment on the thematic matching decisions, perhaps because this task required a more flexible pattern of retrieval suited to the context. However, the SA cases showed slower and less accurate responses across both identity and thematic tasks. This pattern of impairment arose from both frontal and temporoparietal lesions.

Intriguingly, ‘reverse’ specificity effects in identity-matching tasks were only found in frontal patients, and effects of lesion location were greater in the identity task. Superordinate labels activate a broader range of semantic associations than low-frequency specific terms, since they occur in a wider range of contexts. This contextual diversity is thought to increase demands on semantic control, which might be necessary to shape semantic retrieval towards associations relevant for a particular context (Hoffman et al., 2011). Additionally, superordinate labels in this task were repeated more often, as a handful of terms describe a broad range of concepts. It is possible that patients particularly sensitive to re-selecting previously-suppressed distractors were influenced by this repetition, in line with previous findings (Gardner et al., 2012, Thompson et al., 2015). In line with this view, dysexecutive patients with prefrontal damage have shown reverse specificity effects in picture naming tasks (Humphreys and Forde, 2005).

### 6.1 Dual task effects on semantic performance in healthy participants

We next investigated whether the pattern observed in the SA cases could be reproduced under conditions of divided attention in healthy participants. Since the requirement to divide attention between two tasks heavily loads control mechanisms (Baddeley et al., 1997, Della Salla et al., 1995, Logie et al., 2004), such a finding would support the hypothesis that the deficit in SA is related to the control demands of semantic judgements. In healthy volunteers, the possibility that this pattern reflects a selective impairment of thematic representations can also be discounted. We used the same semantic tasks as before, combined with either a relatively automatic or an attention-demanding secondary task during testing (Almaghyuli et al., 2012). We predicted that divided attention would produce a greater disruption to thematic decisions when compared to identity matching in line with our findings from Experiment 1, and that the degree of this disruption would reflect associative strength (i.e. a greater disruptive effect for weak associations compared to strong associations).

## 7 Method

### 7.1 Participants

30 right-handed native English speaking participants (27 females, mean age 20.5 years) were recruited at the University of York. Ethical approval was obtained from the Research Ethics Committee of the Department of Psychology at the University of York. All participants had normal/corrected-to normal vision and were screened for dyslexia through self-report. Two participants (participant 16 and 24) with incomplete datasets were removed from the analysis leaving a total of 28 participants.

### 7.2 Design

The semantic tasks and stimuli were identical to Experiment 1. This study examined performance according to: (1) semantic task (identity vs. associative); (2) semantic task difficulty (strong and weak thematic associations, and specific and superordinate identity); and (3) dual task difficulty (count or 1-back). An easy ‘count’ version of the dual task involved reproducing orally presented numbers in numerical order (e.g., 1 – “1”, 2 – “2”, 3 – “3”). A more demanding 1-back condition required participants to produce the item presented on the *previous* trial (i.e., 1 – “no response”, 6 – “1”, 3 – “6”). Thus, the 1-back condition placed greater demands on working memory, updating and controlled retrieval in the face of potential interference from the most recent input.

### 7.3 Procedure

Participants in this task, unlike Experiment 1, were only given 3000 ms to make their decision, after which the next trial was presented. Participants were also simultaneously engaged in a secondary task, presented, and scored using the N-backer program (Monk et al., 2011). Both the count sequence and 1-back conditions for the secondary task involved spoken numbers every 1.5 s, presented over headphones. Participants had to give a spoken response before the next number was presented. The semantic tasks were presented using E-prime on a second computer and involved visual inputs and manual responses.

At the beginning of the session, participants practiced both the easy and hard secondary task for 30 s in combination with the semantic tasks, allowing participants to become familiar with speed of presentation and the experimental tasks. An experimental session started with a set of 40 practice trials divided equally across thematic and identity judgements, which were performed three times; under single task conditions, during the count sequence task, and during 1-back. Semantic tasks and secondary tasks were blocked with participants completing 60 trials for each semantic judgement type. After 60 trials the participant stopped all tasks in order to read the instruction slide for the next set of semantic judgements. The order of identity and thematic judgements and the order of the secondary tasks were counterbalanced across participants.

## 8 Results

**Fig. 6:**
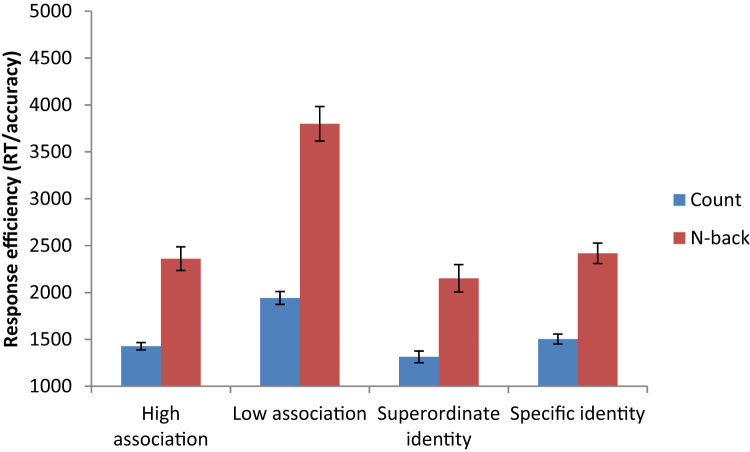
Effects of semantic task, difficulty and secondary task for response efficiency in healthy participants. Error bars represent the standard error of the mean.

Table 11 shows RT and accuracy for both semantic tasks whilst performing the easy (count sequence) and difficult (1-back) secondary tasks, with response efficiency displayed in Fig. 6.

**Table 11:** Healthy controls performance on the semantic tasks whilst performing the secondary tasks.

	Reaction time		Accuracy	
Condition	Mean (milliseconds)	S.D	Mean (proportion correct)	S.D
*Count sequence secondary task*				
High thematic strength	1312.08	167.68	.92	.06
Low thematic strength	1555.50	175.43	.81	.10
Superordinate identity	1180.04	201.08	.91	.09
Specific identity	1289.04	230.82	.86	.07
*1-back secondary task*				
High thematic strength	1609.21	250.84	.72	.16
Low thematic strength	1873.03	214.73	.52	.12
Superordinate identity	1574.90	241.50	.77	.16
Specific identity	1571.28	263.18	.67	.11

### 8.1 Thematic vs. identity judgements

Table 12 shows the contrast between thematic and identity matching. The thematic task was harder than the identity matching task overall. Performance was poorer when the semantic task was combined with the 1-back dual task condition, compared with the count condition. There was also an interaction between dual task and semantic judgement type, such that particularly poor performance was observed in the thematic task when it was paired with the 1-back condition.

**Table 12:** ANOVAs showing difference between tasks.

	Response efficiency (RT/accuracy)		Accuracy		Reaction time	
	F	Sig.	F	Sig.	F	Sig.
Dual task	134.169	< .001	145.149	< .001	97.043	< .001
Judgment	101.083	< .001	43.795	< .001	95.630	< .001
Dual task by judgement	20.681	< .001	17.206	.008	.282	.600

### 8.2 Strength of association

Table 13 shows analysis of the effect of the strength of association within the thematic task. Weak thematic associations were more difficult to retrieve, particularly under dual task conditions, giving rise to a significant interaction between associative strength and dual task in response efficiency and accuracy.

**Table 13:** ANOVAs for strength of association.

	Response efficiency (RT/accuracy)		Accuracy		Reaction time	
	F	Sig.	F	Sig.	F	Sig.
Dual task	119.479	< .001	131.821	< .001	66.336	< .001
Strength	151.799	< .001	111.884	< .001	138.947	< .001
Dual task by strength	27.173	< .001	11.658	.002	.156	.696

### 8.3 Specificity effects

We then compared specific and general-level matching within the identity matching task (see Table 14). There was a significant specificity effect: the specific items were harder than the superordinate items. This time, there were no significant interactions with dual task, although the interaction was approaching significance for RT.

**Table 14:** ANOVAs for specificity.

	Response efficiency (RT/accuracy)		Accuracy		Reaction time	
	F	Sig.	F	Sig.	F	Sig.
Dual task	80.593	< .001	78.985	< .001	68.717	< .001
Specificity	8.214	.008	21.676	< .001	3.136	.088
Dual task by specificity	.328	.571	2.950	.097	3.911	.058

### 8.4 Subset of items matched for difficulty

Given the overall difference in response difficulty for the identity and thematic tasks, we selected a subset of 87/116 items per task which showed matched performance in the easy dual task condition requiring counting. This included the ‘hardest’ identity trials and ‘easiest’ thematic trials, collapsing across specificity and strength of association (48 specific-level and 39 superordinate-level identity-matching items; 57 strong associations and 30 weak associations from the thematic task). Paired-samples *t*-tests confirmed no difference in response efficiency between thematic and identity tasks for the count condition: t(27) = 1.117, p = .274. When combined with the 1-back task, however, there was a significant difference between semantic judgements, where the identity task was more efficient than the thematic task: t(27) = 4.125, p < .001. This is consistent with the view that executive processes, depleted in the 1-back condition, are more critical for the thematic than identity task.

### 8.5 Summary

The requirement to selectively attend to items in working memory (as opposed to producing an over-learned sequence of numbers relatively automatically) disrupted thematic matching more than identity matching, particularly when the associations were weak. Thus, divided attention in healthy participants disrupted the semantic judgements that were most impaired in SA.

## 9 Discussion

In this study, we tested a potential causal link between semantic control and the retrieval of thematic associations, since these aspects of semantic cognition have been previously noted to rely on overlapping brain regions (Davey et al., 2015b). We compared knowledge of thematic associations and object identity in SA patients (Experiment 1) and healthy participants under dual task conditions (Experiment 2). In addition, we manipulated the difficulty of thematic judgements by contrasting strong with weaker associations, and we compared superordinate and specific identity matching tasks. The retrieval of weak associations is expected to require the greatest level of control, as these items require processing to be focussed on non-dominant links and directed away from strong yet irrelevant aspects of knowledge (Badre et al., 2005, Jefferies, 2013, Noonan et al., 2010). In contrast, although we observed some evidence that the retrieval of specific identity is globally harder than the retrieval of superordinate identity because concepts must be specified more precisely, this manipulation might not have the same impact on the requirement for control – indeed, the control demands of the superordinate decisions are arguably higher because the words in these trials had higher contextual diversity (i.e., they had variable meanings across different contexts).

The data support the view that semantic control processes are critical for accessing thematic associations, particularly when these associations are not strongly supported by the structure of long-term semantic knowledge. Thematic links, particularly those which do not align well with previous experience, might require interaction between the semantic store and additional control processes, which are impaired in patients with SA. To retrieve non-dominant thematic links, retrieval must be shaped to focus on currently-relevant aspects of knowledge and task-irrelevant associations have to be suppressed. In this way, non-dominant thematic judgments might require an additional online “goal” to be internally generated based on the current semantic context and then used to constrain the pattern of retrieval. The identity of an item is less variable across contexts, and so identity matching tasks are unlikely to require this control process to the same degree. Our findings from healthy subjects, under dual-task conditions, corroborated with this patient data. We found that healthy controls took longer and were less accurate on weak association judgements, especially when attention was divided. Intriguingly, performance in both thematic and identity tasks was impaired in patients, and in healthy subjects under dual-task conditions - adding evidence against the thematic hub hypothesis.

While the harder, weak-association trials were more disrupted than judgements about strong thematic links in both experiments, the identity task did not show parallel effects of difficulty. Identifying objects at a specific level was generally more difficult (i.e., error rates were higher in young healthy controls, even in the easy condition, and reaction time was longer in the older controls), and specific-level knowledge is especially vulnerable to neurodegeneration in the ATL in semantic dementia (Bozeat et al., 2000, Hoffman et al., 2012, Patterson et al., 2007). Yet the SA patients investigated here sometimes showed a different pattern: some individuals with pronounced deficits, particularly those with frontal lesions, showed *reverse* specificity effects (i.e., more impairment for superordinate labels). This adds to evidence against the ‘thematic hub’ hypothesis, since patients were impaired on this identity task, and difficulty manipulation results appeared to be related to the patients’ severity. Superordinate terms like animal have substantially higher frequency than specific labels like Labrador, and appear in a greater range of contexts – for example, animal might refer to a pet, a zoo animal, a wild animal or a badly behaving human. In contrast, specific terms have specific uses: Labrador is likely to be a pet, and none of the other contexts apply (Hoffman and Lambon Ralph, 2011). This pattern is consistent with previous reports of higher control demands for superordinate-level terms affecting picture naming performance in SA (Crutch and Warrington, 2008) and other patients with semantic impairment in the context of dysexecutive syndrome (Humphreys and Forde, 2005). It was only the most severe SA cases who showed significant reverse specificity effects, while the more mildly impaired cases (overall task accuracy > 90%) showed exaggeration of the normal pattern (i.e., more impairment for specific labels). This magnification may have reflected strong competition between the three possible response items which were drawn from the same category; and this effect may have been particularly strong for milder patients who could identify the correct interpretation of animal at the superordinate level.

The SA patients typically had large frontoparietal lesions affecting regions implicated in action and event understanding (de Zubicaray et al., 2013, Schwartz et al., 2011) and semantic control (Davey et al., 2015b, Davey et al., 2016, Noonan et al., 2013). The majority of our patient sample had damage to both anterior and posterior components of this large-scale distributed network, although a subset had lesions restricted to posterior temporal and parietal regions. We found that both sets of cases showed deficits affecting the retrieval of weak associations, consistent with previous work showing that damage to anterior and posterior components of this network produces qualitatively similar behavioural deficits (Berthier, 2001, Corbett et al., 2011, Jefferies and Lambon Ralph, 2006, Noonan et al., 2010). This finding is also in line with the finding that LIFG and pMTG both show more activation in control-demanding semantic tasks (Davey et al., 2016, Jefferies, 2013, Noonan et al., 2013). Nevertheless, the semantic control deficits in the anterior patients did appear to be more severe than those in the temporoparietal group; further research is needed to establish if this pattern would generalise across samples and tasks. In addition, the patients’ lesions, even when restricted to posterior temporal and parietal regions, potentially encompassed separable components. fMRI and TMS research in healthy participants has shown that two regions within temporoparietal cortex – angular gyrus and posterior middle temporal gyrus – both contribute to event understanding, but in distinct ways (Davey et al., 2015a, Davey et al., 2016): angular gyrus is implicated in the retrieval of strong conceptual combinations and associations (Humphreys and Lambon Ralph, 2015), together with ATL (Davey et al., 2016, Lau et al., 2013), while posterior middle temporal gyrus supports controlled retrieval of weak associations (Badre et al., 2005, Davey et al., 2015a, Davey et al., 2016, Gold et al., 2006, Noonan et al., 2013). The patients’ lesions were focussed in pMTG as opposed to angular gyrus, consistent with the pattern we observed.

In conclusion, these data support the view that the neurocognitive processes underlying semantic control and thematic associations are overlapping because of a particular requirement for flexible, context-driven retrieval in judging weak thematic relationships. SA patients have greater deficits in thematic than identity judgements, particularly when the target association is relatively weak. When task-relevant information is strongly encoded within the long-term semantic store (i.e., for strong associations), there is little need to shape patterns of retrieval to perform the task. In contrast, for weaker associations, semantic control processes can identify and promote relevant linking features within the semantic store, so that these form a coherent pattern of semantic retrieval. Regions in both PFC and posterior temporal cortex appear to be causally implicated in this aspect of understanding event or thematic associations.

## References

[bib1] Almaghyuli A., Thompson H.E., Lambon Ralph M.A., Jefferies E. (2012). Deficits of semantic control produce absent or reverse frequency effects in comprehension: evidence from neuropsychology and dual task methodology. Neuropsychologia.

[bib2] Baddeley A., Della Salla S., Papagno C., Spinnler H. (1997). Dual-task performance in dysexecutive and nondysexecutive patients with a frontal lesion. Neuropsychology.

[bib3] Badre D., Poldrack R.A., Paré-Blagoev E.J., Insler R.Z., Wagner A.D. (2005). Dissociable controlled retrieval and generalized selection mechanisms in ventrolateral prefrontal cortex. Neuron.

[bib4] Bemis D.K., Pylkkanen L. (2011). Simple composition: a magnetoencephalography investigation into the comprehension of minimal linguistic phrases. J. Neurosci..

[bib5] Berthier M.L. (2001). Unexpected brain-language relationships in aphasia: evidence from transcortical sensory aphasia associated with frontal lobe lesions. Aphasiology.

[bib6] Binder J.R., Desai R.H. (2011). The neurobiology of semantic memory. Trends Cogn. Sci..

[bib7] Binney R.J., Embleton K.V., Jefferies E., Parker G.J.M., Lambon Ralph M.A. (2010). The ventral and inferolateral aspects of the anterior temporal lobe are crucial in semantic memory: evidence from a novel direct comparison of distortion-corrected fMRI, rTMS, and semantic dementia. Cereb. Cortex.

[bib8] Bozeat S., Lambon Ralph M.A., Patterson K., Garrard P., Hodges J.R. (2000). Non-verbal semantic impairment in semantic dementia. Neuropsychologia.

[bib9] Burgess P.W., Shallice T. (1997). The Hayling and Brixton Tests.

[bib10] Coltheart M. (1981). The MRC psycholinguistic database. Q. J. Exp. Psychol. Sect. A.

[bib11] Corbett F., Jefferies E., Ehsan S., Lambon Ralph M.A. (2009). Different impairments of semantic cognition in semantic dementia and semantic aphasia: evidence from the non-verbal domain. Brain.

[bib12] Corbett F., Jefferies E., Lambon Ralph M.A. (2009). Exploring multimodal semantic control impairments in semantic aphasia: evidence from naturalistic object use. Neuropsychologia.

[bib13] Corbett F., Jefferies E., Lambon Ralph M.A. (2011). Deregulated semantic cognition follows prefrontal and temporoparietal damage: evidence from the impact of task constraint on non-verbal object use. J. Cogn. Neurosci..

[bib14] Crawford J.R., Garthwaite P.H. (2002). Investigation of the single case in neuropsychology: confidence limits on the abnormality of test scores and test score differences. Neuropsychologia.

[bib15] Crawford J.R., Garthwaite P.H., Porter S. (2010). Point and interval estimates of effect sizes for the case-controls design in neuropsychology: rationale, methods, implementations, and proposed reporting standards. Cogn. Neuropsychol..

[bib16] Crutch S.J., Warrington E.K. (2008). Contrasting patterns of comprehension for superordinate, basic-level, and subordinate names in semantic dementia and aphasic stroke patients. Cogn. Neuropsychol..

[bib17] Damasio H., Damasio A.R. (1989). Lesion Analysis in Neuropsychology.

[bib18] Davey J., Cornelissen P.L., Thompson H.E., Sonkusare S., Hallam G., Smallwood J., Jefferies E. (2015). Automatic and controlled semantic retrieval: TMS reveals distinct contributions of posterior middle temporal gyrus and angular gyrus. J. Neurosci..

[bib19] Davey J., Rueschemeyer S.A., Costigan A., Murphy N., Krieger-Redwood K., Hallam G., Jefferies E. (2015). Shared neural processes support semantic control and action understanding. Brain Lang..

[bib20] Davey J., Thompson H.E., Hallam G., Karapanagiotidis T., Murphy C., De Caso I., Jefferies E. (2016). Exploring the role of the posterior middle temporal gyrus in semantic cognition: integration of anterior temporal lobe with executive processes. NeuroImage.

[bib21] Davis C.J. (2005). N-watch: a program for deriving neighborhood size and other psycholinguistic statistics. Behav. Res. Methods.

[bib22] de Zubicaray G.I., Hansen S., McMahon K.L. (2013). Differential processing of thematic and categorical conceptual relations in spoken word production. J. Exp. Psychol. Gen..

[bib23] Della Salla S., Baddeley A., Papagno C., Spinnler H. (1995). Dual-task paradigm: a means to examine the central executive. Ann. N. Y. Acad. Sci..

[bib24] Dumais S.T. (2005). Latent semantic analysis. Annu. Rev. Inf. Sci. Technol..

[bib25] Gardner H.E., Lambon Ralph M.A., Dodds N., Jones T., Eshan S., Jefferies E. (2012). The differential contributions of pFC and temporoparietal cortices to multimodal semantic control: exploring refractory effects in semantic aphasia. J. Cogn. Neurosci..

[bib26] Gold B.T., Balota D.A., Jones S.J., Powell D.K., Smith C.D., Andersen A.H. (2006). Dissociation of automatic and strategic lexical-semantics: functional magnetic resonance imaging evidence for differing roles of multiple frontotemporal regions. J. Neurosci..

[bib27] Hodges J.R., Patterson K. (2007). Semantic dementia: a unique clinicopathological syndrome. Lancet Neurol..

[bib28] Hoffman P., Jones R.W., Lambon Ralph M. (2013). Be concrete to be comprehended: consistent imageability effects in semantic dementia for nouns, verbs, synonyms and associates. Cortex.

[bib29] Hoffman P., Jones R.W., Lambon Ralph M.A. (2012). The degraded concept representation system in semantic dementia: damage to pan-modal hub, then visual spoke. Brain.

[bib30] Hoffman P., Lambon Ralph M.A. (2011). Reverse concreteness effects are not a typical feature of semantic dementia: evidence for the Hub-and-Spoke model of conceptual representation. Cereb. Cortex.

[bib31] Hoffman P., Rogers T.T., Lambon Ralph M.A. (2011). Semantic diversity accounts for the “missing” word frequency effect in stroke aphasia: insights using a novel method to quantify contextual variability in meaning. J. Cogn. Neurosci..

[bib32] Humphreys G.F., Lambon Ralph M.A. (2015). Fusion and fission of cognitive functions in the human parietal cortex. Cereb. Cortex.

[bib33] Humphreys G.W., Forde E.M.E. (2005). Naming a giraffe but not an animal: base-level but not superordinate naming in a patient with impaired semantics. Cogn. Neuropsychol..

[bib34] Jackson R.L., Hoffman P., Pobric G., Lambon Ralph M. (2015). The nature and neural correlates of semantic association versus conceptual similarity. Cereb. Cortex.

[bib35] Jefferies E. (2013). The neural basis of semantic cognition: converging evidence from neuropsychology, neuroimaging and TMS. Cortex.

[bib36] Jefferies E., Baker S.S., Doran M., Lambon Ralph M.A. (2007). Refractory effects in stroke aphasia: a consequence of poor semantic control. Neuropsychologia.

[bib37] Jefferies E., Lambon Ralph M.A. (2006). Semantic impairment in stroke aphasia versus semantic dementia: a case-series comparison. Brain.

[bib38] Jefferies E., Patterson K., Jones R.W., Lambon Ralph M.A. (2009). Comprehension of concrete and abstract words in semantic dementia. Neuropsychology.

[bib39] Jefferies E., Patterson K., Lambon Ralph M.A. (2008). Deficits of knowledge versus executive control in semantic cognition: insights from cued naming. Neuropsychologia.

[bib40] Kalénine S., Mirman D., Middleton E.L., Buxbaum L.J. (2012). Temporal dynamics of activation of thematic and functional knowledge during conceptual processing of manipulable artifacts. J. Exp. Psychol.: Learn. Mem. Cogn..

[bib41] Kalénine S., Mirman D., Middleton E.L., Buxbaum L.J. (2013). Temporal dynamics of activation of thematic and functional knowledge during conceptual processing of manipulable artifacts. J. Exp. Psychol. Learn. Mem. Cogn..

[bib42] Kalénine S., Peyrin C., Pichat C., Segebarth C., Bonthoux F., Baciu M. (2009). The sensory-motor specificity of taxonomic and thematic conceptual relations: a behavioral and fMRI study. NeuroImage.

[bib43] Kotz S. (2002). Modulation of the Lexical–Semantic network by auditory semantic priming: an event-related functional MRI study. NeuroImage.

[bib44] Lambon Ralph M., Jefferies E., Patterson K., Rogers T.T. (2017). The neural and computational bases of semantic cognition. Nat. Rev. Neurosci..

[bib45] Landauer T.K., Foltz P.W., Laham D. (1998). Introduction to latent semantic analysis. Discourse Process..

[bib46] Lau E.F., Gramfort A., Hämäläinen M.S., Kuperberg G.R. (2013). Automatic semantic facilitation in anterior temporal cortex revealed through multimodal neuroimaging. J. Neurosci..

[bib47] Logie R.H., Cocchini G., Della Salla S., Baddeley A. (2004). Is there a specific executive capacity for dual task coordination? Evidence from Alzheimer's disease. Neuropsychology.

[bib48] Martin A. (2007). The representation of object concepts in the brain. Annu. Rev. Psychol..

[bib49] Mirman D., Graziano K.M. (2012). Individual differences in the strength of taxonomic versus thematic relations. J. Exp. Psychol. Gen..

[bib50] Monk A.F., Jackson D., Nielsen D., Jefferies E., Olivier P. (2011). N-backer: an auditory n-back task with automatic scoring of spoken responses. Behav. Res. Methods.

[bib51] Moss H.E., Rodd J.M., Stamatakis E.A., Bright P., Tyler L.K. (2005). Anteromedial temporal cortex supports fine-grained differentiation among objects. Cereb. Cortex.

[bib52] Mummery C.J., Patterson K., Price C.J., Ashburner J., Frackowiak R.S.J., Hodges J.R. (2000). A voxel-based morphometry study of semantic dementia: relationship between temporal lobe atrophy and semantic memory. Ann. Neurol..

[bib53] Noonan K.A., Jefferies E., Corbett F., Lambon Ralph M.A. (2010). Elucidating the nature of deregulated semantic cognition in semantic aphasia: evidence for the roles of prefrontal and temporo-parietal cortices. J. Cogn. Neurosci..

[bib54] Noonan K.A., Jefferies E., Visser M.E.J., Lambon Ralph M.A. (2013). Going beyond inferior prefrontal involvement in semantic control: evidence for the additional contribution of parietal and posterior middle temporal cortex. J. Cogn. Neurosci..

[bib55] Noppeney U.T.A. (2008). The neural systems of tool and action semantics: a perspective from functional neuroimaging. J. Physiol..

[bib56] Patterson K., Nestor P.J., Rogers T.T. (2007). Where do you know what you know? The representation of semantic knowledge in the human brain. Nat. Rev. Neurosci..

[bib57] Phan T.G., Donnan G.A., Wright P.M., Reutens D.C. (2005). A digital map of middle cerebral artery infarcts associated with middle cerebral artery trunk and branch occlusion. Stroke.

[bib58] Phan T.G., Fong A.C., Donnan G.A., Reutens D.C. (2007). Digital map of posterior cerebral artery infarcts associated with posterior cerebral artery trunk and branch occlusion. Stroke.

[bib59] Pobric G., Jefferies E., Lambon Ralph M.A. (2010). Amodal semantic representations depend on both anterior temporal lobes: evidence from repetitive transcranial magnetic stimulation. Neuropsychologia.

[bib60] Raven J.C. (1962). Coloured Progressive Matrices Sets A, AB, B.

[bib61] Reitan R.M. (1958). Validity of the Trail Making test as an indicator of organic brain damage. Percept. Mot. Skills.

[bib62] Robertson I.H., Ward T., Ridgeway V., Nimmo-Smith I. (1994). The Test of Everyday Attention.

[bib63] Rogers T.T., Hocking J., Noppeney U.T.A., Mechelli A., Gorno-Tempini M.L., Patterson K., Price C.J. (2006). Anterior temporal cortex and semantic memory: Reconciling findings from neuropsychology and functional imaging. Cogn. Affect. Behav. Neurosci..

[bib64] Rogers T.T., Lambon Ralph M.A., Garrard P., Bozeat S., McClelland J.L., Hodges J.R., Patterson K. (2004). Structure and deterioration of semantic memory: a neuropsychological and computational investigation. Psychol. Rev..

[bib65] Rogers T.T., Patterson K., Jefferies E., Lambon Ralph M.A. (2015). Disorders of representation and control in semantic cognition: effects of familiarity, typicality, and specificity. Neuropsychologia.

[bib66] Sachs O., Weis S., Krings T., Huber W., Kircher T. (2008). Categorical and thematic knowledge representation in the brain: neural correlates of taxonomic and thematic conceptual relations. Neuropsychologia.

[bib67] Samson D., Connolly C., Humphreys G.W. (2007). When "happy" means "sad": neuropsychological evidence for the right prefrontal cortex contribution to executive semantic processing. Neuropsychologia.

[bib68] Sass K., Sachs O., Krach S., Kircher T. (2009). Taxonomic and thematic categories: neural correlates of categorization in an auditory-to-visual priming task using fMRI. Brain Res..

[bib69] Schwartz M.F., Kimberg D.Y., Walker G.M., Brecher A., Faseyitan O.K., Dell G.S., Coslett H.B. (2011). Neuroanatomical dissociation for taxonomic and thematic knowledge in the human brain. Proc. Natl. Acad. Sci..

[bib70] Seghier M.L., Ramlackhansingh A., Crinion J., Leff A.P., Price C.J. (2008). Lesion identification using unified segmentation-normalisation models and fuzzy clustering. NeuroImage.

[bib71] Solène K., Buxbaum L.J. (2016). Thematic knowledge, artifact concepts, and the left posterior temporal lobe: Where action and object semantics converge. Cortex.

[bib72] Thompson H.E., Robson H., Lambon Ralph M.A., Jefferies E. (2015). Varieties of semantic 'access' deficit in Wernicke's Aphasia and Semantic Aphasia. Brain.

[bib73] Van Casteren M., Davis M.H. (2007). Match: a program to assist in matching the conditions of factorial experiments. Behav. Res. Methods.

[bib74] Visser M.E.J., Jefferies E., Embleton K.V., Lambon Ralph M.A. (2012). Both the middle temporal gyrus and the ventral anterior temporal area are crucial for multimodal semantic processing: distortion-corrected fMRI evidence for a double gradient of information convergence in the temporal lobes. J. Cogn. Neurosci..

[bib75] Warrington E.K., James M. (1991). The Visual Object and Space Perception Battery.

[bib76] Wechsler D. (1987). Wechsler Memory Scale - Revised (WMS-R).

[bib77] Whitney C., Kirk M., O'Sullivan J., Lambon Ralph M.A., Jefferies E. (2011). The neural organization of semantic control: TMS evidence for a distributed network in left inferior frontal and posterior middle temporal gyrus. Cereb. Cortex.

